# Learning to explore the structure of kinematic objects in a virtual environment

**DOI:** 10.3389/fpsyg.2015.00374

**Published:** 2015-04-07

**Authors:** Marcus Buckmann, Robert Gaschler, Sebastian Höfer, Dennis Loeben, Peter A. Frensch, Oliver Brock

**Affiliations:** ^1^Department of Psychology, Humboldt-UniversitätBerlin, Germany; ^2^Center for Adaptive Behavior and CognitionMax Planck Institute for Human Development, Germany; ^3^Department of Psychology, Universität Koblenz-LandauLandau, Germany; ^4^Department of PsychologyFernUniversität in Hagen, Germany; ^5^Robotics and Biology Laboratory, Technische Universität BerlinBerlin, Germany

**Keywords:** object exploration, skill acquisition, virtual environment, strategy selection

## Abstract

The current study tested the quantity and quality of human exploration learning in a virtual environment. Given the everyday experience of humans with physical object exploration, we document substantial practice gains in the time, force, and number of actions needed to classify the structure of virtual chains, marking the joints as revolute, prismatic, or rigid. In line with current work on skill acquisition, participants could generalize the new and efficient psychomotor patterns of object exploration to novel objects. On the one hand, practice gains in exploration performance could be captured by a negative exponential practice function. On the other hand, they could be linked to strategies and strategy change. After quantifying *how much* was learned in object exploration and identifying the time course of practice-related gains in exploration efficiency (speed), we identified *what* was learned. First, we identified strategy components that were associated with efficient (fast) exploration performance: sequential processing, simultaneous use of both hands, low use of pulling rather than pushing, and low use of force. Only the latter was beneficial irrespective of the characteristics of the other strategy components. Second, we therefore characterized efficient exploration behavior by strategies that simultaneously take into account the abovementioned strategy components. We observed that participants maintained a high level of flexibility, sampling from a pool of exploration strategies trading the level of psycho-motoric challenges with exploration speed. We discuss the findings pursuing the aim of advancing intelligent object exploration by combining analytic (object exploration in humans) and synthetic work (object exploration in robots) in the same virtual environment.

## Introduction

For humans (e.g., Vaesen, 2012) as well as for robots (Höfer et al., 2014) exploration of the kinematic structure of objects is a pre-requisite for tool use. While both can precisely perform complicated movement patterns (Pfeifer et al., 2012; Verrel et al., 2013), relatively little is known about efficient strategies for exploring the kinematic properties of objects in either case. A combination of an analytic (humans) and synthetic (robot experiment) approach can provide an efficient means to study intelligent object exploration. However, this requires a common test-bed for human and robot object exploration, as well as means to qualitatively and quantitatively analyze and describe human exploration behavior. Focusing on the latter, we present first steps to develop such an approach in a virtual environment similar to the one used in the robot simulation studies of Katz et al. (2008).

Humans can interact easily and accurately with objects, because they explore their structure and features very efficiently (Klatzky et al., 1985). The ability to perform such exploratory behavior constitutes an important component of humans' ability to adapt to significant variability in the environment. Of course, competent object use is driven in part by knowledge about the object's structure and function (Rosch et al., 1976; Lederman and Klatzky, 1990, 2004). For instance, a human familiar with scissors will not need a long time to explore and use the structure of gas pipe pliers, since kinematic structure and function are similar. However, efficient exploration strategies might be at least as important for efficient object exploration, as precise sensors and effectors. For example, an assistance robot should use minimal time and a low number of interactions in order to explore the most crucial aspects of an object that could potentially serve as a tool. Humans already exhibit this capability and can adapt to new environments and tasks flexibly and effectively. We therefore believe that the analysis of human exploration behavior will not only lead to an understanding of human behavior but will also enable exploration behavior in a novel generation of robots that learn from interactions with their environment.

Exploration problems are often presented to humans as symbolic problem solving tasks. These tasks place minimal demands on physical interaction with the environment so that neither its potential costs nor its potential scaffolding function in the problem solving process can be evaluated (e.g., Wason, 1960; Gaschler et al., 2012; Wakebe et al., 2012). Note that in some cases, symbolic problem solving has been combined with elaborate physical interaction (e.g., Klahr and Dunbar, 1988). However, flexible variation of experimental factors of an exploration task in a physical environment is difficult to achieve. In contrast, virtual environments effortlessly afford such variability and also enable the recording of force-interaction data. In virtual environments the experimenter can, for instance, flexibly arrange rooms to explore (Williams et al., 2007) and experimental control can involve behavior-contingent manipulations that can only be achieved in a virtual environment. For instance, Patsenko and Altmann (2010) exchanged virtual discs of the Tower of Hanoi problem solving task contingently upon eye-movements in order to probe adherence to advance planning vs. *ad-hoc* re-planning. Participants adapted their plans for the next problem solving steps in line with the (predominantly unnoticed) changes in the virtual environment. Apart from attractive options in data logging and experimental manipulations, virtual environments allow for massed training before or after exposure to test situations outside the virtual environment (e.g., Monge Pereira et al., 2014). Challenge level can be fine-tuned to the needs of special populations, and training can involve situations that would be too infrequent, too dangerous or too expensive to train outside the virtual environment. For instance, researchers have reported transfer of motor training to test measures (e.g., Monge Pereira et al., 2014), as well induction of spatial orientation by vision and movement (e.g., Williams et al., 2007) or by haptics (e.g., Afonso et al., 2010) in virtual environments. Most important for the current research, virtual environments allow to enrich problem solving tasks by psycho-motoric demands. Different from many learning tasks involving exploration (cf. Gaissmaier and Schooler, 2008; Gaschler et al., 2014a), stimuli are complex, come in varied form and change dynamically according to participants' actions. Data logging in the virtual environment allows to keep track of the dynamically changing stimuli and actions, allowing to analyze changes in exploration behavior across trials. With a virtual environment, the same exploration task can be offered to different research participants. In addition, on the long run, exploration behavior can even be compared between humans and simulated robots operating in the same virtual environment.

Recent work in human movement research (e.g., Verrel et al., 2013) and robotics (e.g., Deimel et al., 2013) suggests that physical constraints inherent in physical interaction with the environment can be used to foster adaptive behavior. However, as of yet this notion is not well represented in work on human skill acquisition and problem solving—two potential components of efficient object exploration skills. Human skill acquisition is often studied either with focus on psychomotor demands (Newell et al., 2001) or with focus on cognitive demands (e.g., Logan, 1988; Gaschler et al., 2015)—rather than with focus on the combination of psychomotor and cognitive demands. In contrast, work on human problem solving has stressed that problem solving can profit from grounding in physical interaction (Watson, 1920; Kirsh, 1995; Vallée-Tourangeau et al., 2011; Werner and Raab, 2013). The arguments presented above suggest that exploration of the kinematic structure of objects should be studied from a learning perspective in a virtual task environment that provides both cognitive and psychomotor challenges. Such a setup offers opportunities and challenges for learning of efficient exploration behavior that would be masked when focusing either on the cognitive or the psychomotor aspects in isolation. Moment-to-moment variation in psychomotor demands influences the amount of cognitive resources available for other tasks and vice versa (e.g., Verrel et al., 2009). Importantly, variability in psychomotor demands can help to avoid that learning processes incorporate arbitrary motor contingencies (cf. Butz et al., 2007). For instance, research on category learning has documented surprising bindings between cognitive learning processes and arbitrary perceptual and motoric demands of the interface. Ashby et al. (2003) observed a striking encapsulation of categorization knowledge. After learning how to differentiate stimuli according to a single-dimensional rule, participants could transfer the acquired categorization knowledge to a setup where the fingers pushing the response buttons changed. However, transfer was not possible if the categorization task demanded the weighing of features on two dimensions, rather than a one-dimensional cutoff rule. While participants were able to acquire the knowledge necessary to classify objects based on a two-dimensional separation criterion, they could no longer use this (supposedly non-verbal) categorization knowledge when the assignment of fingers to response keys was changed.

## The present study

Instructing participants to repeatedly classify the kinematic structure of chains in a virtual environment, the current study tested the quantity and quality of human exploration learning. First we tested to what extent people would increase exploration efficiency (in terms of speed and force) with practice—despite that they can count on many object exploration episodes from outside the lab. Second we tested whether practice would lead to changes in the exploration strategies selected. This could be reflected in a reduction of strategy repertoire vs. maintained flexibility in selecting from a large pool.

**Figure 1:**
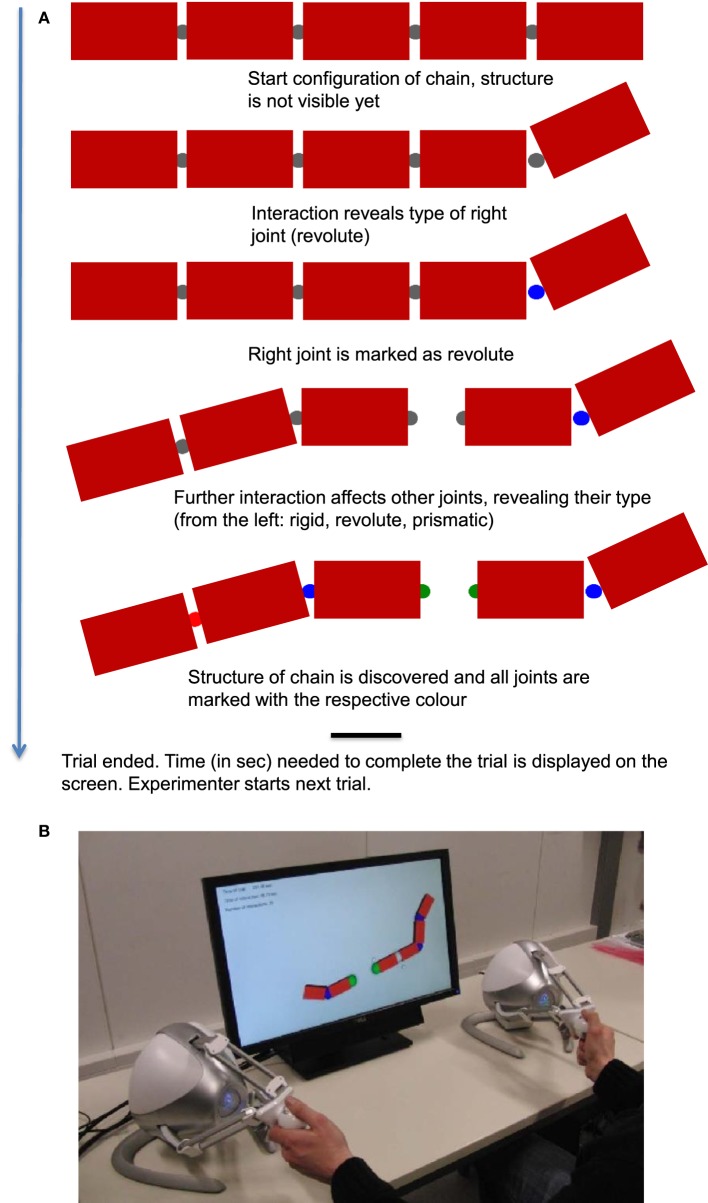
**(A)** shows a schematic description of events in one exploration trial. **(B)** depicts the haptic interface used for the experiment.

Participants had the task to physically manipulate chains in a virtual environment (Figures 1A,B) in order to classify the kinematic structure of each of these chains (i.e., by which type of joints the links of a chain were connected to one another). Everyday observation suggests that people seem to playfully explore the kinematic structure of objects and use them as tools (e.g., decomposing a new type of pen while talking on the phone). Here we tested the extent and kind of learning gains of adults in efficiency of object exploration despite such prior practice. We quantified the practice gains in time, force, and number of actions needed to classify the structure of virtual chains by marking the joints as revolute (i.e., hinge), prismatic (i.e., slider/spring), or rigid (i.e., fixed). With respect to quantity of learning, our research question was whether we could capture practice-related gains in exploration efficiency in a simple mathematical function tied to skill acquisition theories. While such an approach has proven useful in laboratory research on strategy change in skill acquisition when employing tasks with low psycho-motor demands (cf. Anderson, 1982, 2002; Gaschler et al., 2015), learning outside the lab has not always been accessible to such modeling (cf. Gaschler et al., 2014b). It is thus an open question whether mathematical modeling can help to summarize exploration learning in a complex task posing cognitive and psycho-motor challenges.

Apart from testing how much is learned and in what time-course, we probed *what* is learned. On the one hand, we investigated whether gains in exploration efficiency are based on specific memories of prior exploration episodes (cf. Logan, 1988), or, alternatively on the formation of more general exploration strategies (cf. Gaschler et al., 2015). The latter would allow participants to transfer exploration knowledge across chains of different size and shape. On the other hand, we aimed at pinning down exploration strategies. For this we investigated whether components of exploration behavior could be captured by quantitative indicators and whether these components could be traced back to overarching strategies. With practice, participants might optimize the selection of exploration strategies—potentially at the cost of maintaining a diverse repertoire of flexible exploration strategies. In a nutshell, our goal was to quantitatively describe exploration behavior and identify patterns related to efficient exploration. By extracting general features of efficient exploration, we tried to gain a general understanding of what works/does not work for humans so that later work can try to implement parts of these behavioral patterns in robots.

We tested the exploration behavior on randomly generated chains in a human sample (*N* = 19). Similar to Gaschler et al. (2012), the task was to identify the joints of the chain, but this time by physical exploration rather than by keyboard-commanded discrete tests. The structure of the chain was explored by exerting force at blocks or joints of the chain by pulling or pushing (Figure 1A). Interacting with the chain at or close to one link could also move or distort other parts of the chain—the more remote from the point of contact, the less the influence. The movement and position of the links allowed participants to reveal of which type a specific joint was. The haptic interfaces (Figure 1B) provided force feedback to the user, so that the exploration would appear more realistic and intuitive. Both haptic devices (one for the left and one for the right hand) could be used simultaneously.

### Criteria for learning

As we wanted to determine how humans learn to explore the kinematic structures, we needed a criterion for learning. The simulation software of the virtual environment recorded the time which was needed to classify the type of all joints in a chain, registered all interactions with links and recorded expenditure of force. We expected a strong learning effect, denoted by a decrease of the exploration time per chain over the experiment. Apart from recording how much was learned, we wanted to characterize what was learned. One means to characterize learning is to test if this learning is bound to particular material trained or generalizes to variants not practiced or practiced less often (cf. Kramer et al., 1991; Katz et al., 2008; Gaschler et al., 2012). For instance, Katz and Brock (2008) found that a simulated robot could transfer 30% of the gain in speed of exploration from a shorter chain (it was trained on) to a longer chain and Gaschler et al. (2012) found that once a redundancy in the task material is discovered, it is transferred to novel stimuli.

### Identifying interaction strategies

In the current study, we identified general interaction strategies. These interaction strategies were examined on two different levels: the *strategy* level and the *component* level. In order to analyze *strategy components*, each interaction trace in the logged data was analyzed on its own: The amount of force used on the haptic interfaces, the usage of hands (left vs. right vs. both hands simultaneously), the preferred interaction type (pushing vs. pulling the kinematic structure) and the organization of the exploration (sequential interaction vs. unsystematic interaction) are crucial components. These components could be evaluated with regard to frequency of occurrence in the sample and efficiency. Based on this we analyzed combinations of components (i.e., strategy level).

## Methods

### Participants

We tested 19 participants (12 female, *mean age* 24.7 years, *range:* 19–30, *SD* = 3.85, all right handed) from the participant pool of the department of psychology of Humboldt-Universität zu Berlin. Data collection took place in the Robotics and Biology Laboratory at Technical University Berlin. Participants received course credit or € 8 as compensation for participating in the experiment lasting 45–60 min.

### Materials and apparatus

On each trial participants were faced with the task to explore and mark the structure of a kinematic chain. They used two Novint Falcon haptic devices for physical exploration in the virtual environment of 9.8 × 9.8 × 9.8 cm with a position resolution of 400 dpi on a 24-inch LCD screen and force feedback capabilities of 2 lbs controlled by physics engine NVIDIA PhysX Version 2.8.3 on a Linux platform. OpenSceneGraph 3.0 was used for visualization, while the simulation environment was programmed with C++. The visual perspective in the virtual environment was fixed to top view, so that participants had the experience of a two-dimensional setting. The cursors of the haptic interfaces were rigid gray hands with the tips of the virtual index fingers as contact points. All links of the chains had the same form and size (2.4 × 1.3 cm) and were presented in red. The joints were displayed as gray balls at the beginning of each trial and colored specific to the joint type by the participants. Participants used one button of the haptic device for marking the joint types. Another button was reserved for pulling the chains.

To mark a joint, the haptic device had to be moved close to the gap between the links before pressing one marking button. The color of the joint changed with each pressing of the button to indicate the joint type currently selected. The allocation of color to joint type was displayed in the bottom left of the screen (prismatic = green, rigid = red, revolute = blue). When a trial was completed by the correct marking of all joints, the time needed to complete the trial appeared on the screen in green letters. To proceed to the next trial, the experimenter had to press the space bar.

We assigned all joint types (revolute, prismatic, rigid) randomly with equal probability with one exception. For some participants, the 2nd joint from the left was always revolute. Such a redundancy was discovered by the participants in our earlier work with shorter chains and a simplistic interaction mode (Gaschler et al., 2012). Here, we wanted to test whether this would extent to situations with longer structures and enriched interaction opportunities.

### Procedure

Prior to the experiment, participants received an instruction sheet, informing them about the handling of the haptic interface and their task of identifying the joints. Participants underwent a training to get used to control the haptic interfaces under the supervision of the experimenter. In this warm up phase, three boxes had to be moved into a cell on the right side of the screen. This task could only be completed if pushing and pulling behavior were used to navigate the boxes. For pushing, the index finger of the hand-shaped cursor had to be moved against a block in the virtual environment. For pulling, the cursor was moved away while pressing a button at the handle of the haptic interface. After training, the experimenter started the main experiment and later refrained from commenting on the performance of the participants.

On each trial, one chain of 6–9 links (thus, 5–8 joints) was presented. Each participant had to run through four training blocks of eight trials to complete the experiment. We manipulated two variables on two levels, frequency of long chains (length-set condition) and simplification condition. Half of the participants explored 12 chains each of Length 7 and 8 and four chains each of Length 5 and 6 in each of the four training blocks. Reversely, for the other participants the short chains were frequent and the long chains infrequent. Orthogonal to the frequency manipulation we varied between participants whether or not there was an opportunity for a shortcut. In the simplification condition, all chains of the first three blocks had the regularity that the second joint from the left was of the revolute type. Participants were not told about the regularity and randomly assigned to the conditions.

While the experiment was running, the status of the haptic devices was logged with a frequency of 3 Hz. After the exploration of the chains was completed, participants were interviewed concerning whether they had recognized a regularity in the kinematic structures.

### Experimental design

Dependent variables were the time participants needed to complete a trial, the physical force they used on the haptic interface and the interaction type (pushing vs. pulling). Within subjects factors were practice—indexed either in terms of training Block (1–4) or in terms of trials (1–32), as well as the length of the chain (5–8 joints). Between-subjects variables were the manipulations of length set (short chains frequent vs. long chains frequent) and simplification option (shortcut possible vs. not possible). Due to technical difficulties we lost data of one participant in the cell of the design with no simplification condition and high frequency of long chains, leaving four participants in this cell and five in each of the other cells of the two-by-two design. In all analysis reported here, the significance level was set to α = 0.05. The reported ANOVAs were Greenhouse-Geisser corrected.

## Results

Our major goal was to analyze how participants explored the kinematic structures and how exploration changed with practice. Below, we first we characterize practice-related gains in exploration efficiency (speed) according to how learning generalized across different virtual chains. Second, moving beyond this simple ANOVA approach to test transfer, we used mathematical modeling to compare the practice functions derived from different theories on human skill acquisition with the time course of improvements on the group level as well as the level of individual participants. Third, we developed measures to identify components of exploration strategies and related these components to exploration efficiency via hierarchical regression. Last we combined different components to strategies differing in the exploration efficiency they bring about.

### Quantifying practice related gains and transfer across chains

**Figure 2:**
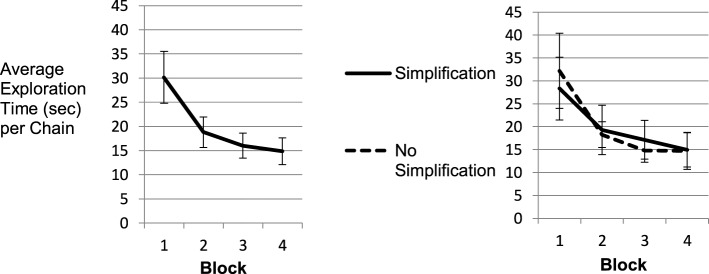
**Training effect in exploration performance**. The left panel shows the average time participants needed to complete exploration on one chain. The right chart depicts the same relation with separated groups of simplification vs. no simplification condition.

Figure 2 (left panel) shows that average time demand per successful exploration decreased by 50% from 30 to 15 s per chain from Block 1 (Trials 1–8) to Block 4 (Trials 25–32). While the left panel depicts the data averaged across all participants, in the right panel two groups of participants were analyzed separately: participants exposed to chains with joints selected fully at random vs. participants for whom the second joint from left was revolute in Blocks 1–3 (and random in Block 4). This provided participants with the opportunity to directly mark the fixed joint if they had learned about the regularity.

While prior results of tasks placing little load on psychomotor control showed substantial shortcut usage based on the discovery of simple regularities (Gaschler et al., 2012), this was not the case in the current setup with long chains, two-handed operation and kinematic feedback. The right panel of the graph does not suggest any differences in exploration time for participants with vs. without option for simplification. Accordingly, a mixed repeated measures analysis of variance (ANOVA) with Block as within participants factor and simplification option as a between participants factor only showed a main effect of Block, *F*_(1.44, 24.59)_ = 42.38, *MSE* = 46.21, *p* < 0.001, η*p^2^* = 0.714. There was no effect of simplification option (*F* = 0), nor any interaction effect. In line with the lack of a behavioral effect, the debriefing interview revealed that no participant in the simplification condition (*N* = 10) was aware of the regularity in the chain structure. Consistent with the absent difference in exploration time between the two groups, participants were not slowed down either, when in Block 4 the regularity in Joint 2 did no longer hold (Figure 2, right panel).

As explained above, the experiment contained a second manipulation in order to probe the nature of the learning underlying the substantial improvement in performance we observed. Apart from varying whether or not the joint type of one joint was predictable, we varied the frequency with which chains of different length were presented. If learning was in part specific to chain length (cf. Katz and Brock, 2008), participants should explore chains of the length they are exposed to most frequently faster as compared to chains of the less frequent length. To the extent however, that learning generalizes across chains of different length, there should not be an effect of training frequency. Furthermore, there could in principle be an overall advantage of participants exposed to the long chains frequently. This is because long chains contain more joints and in consequence, the participants exploring many long chains had more exposure to joints during the 32 trials performed in the task.

**Figure 3:**
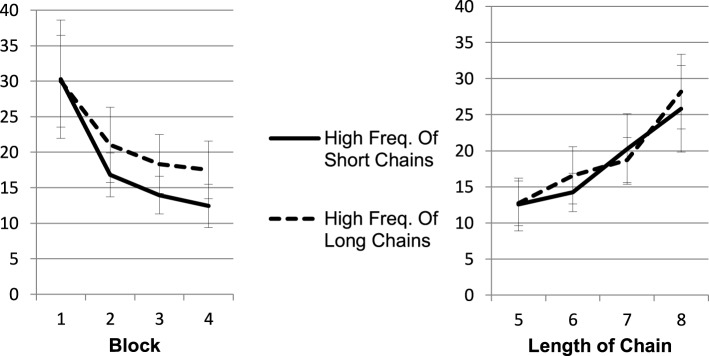
**Average exploration time depending on the frequency of the chain types (short chains frequently vs. long chains frequently)**. The left panel depicts that participants in the condition with high frequency of long chains on average needed more time for exploration. As the right panel pictures, this effect is exclusively explained by the larger amount of longer chains contributing to the average. There are no differences between the length-set groups in the speed of exploring the chains of different lengths.

The results (Figure 3) suggest that learning was not specific to chain length and was based on trials (exploring all joints of one entire chain) rather than on number of joints per trial. Participants exploring many long chains were not faster on long chains than participants with many short and few long chains. A mixed ANOVA verified the impression of Figure 3. Speedup across blocks led to a main effect of block, *F*_(1.21, 20.48)_ = 37.43, *MSE* = 718.86, *p* < 0.001, η^2^_*p*_ = 0.69. As trials with longer chains led to longer exploration times as compared to trials with shorter chains, we obtained a main effect of length, *F*_(2.20, 37.53)_ = 14.90, *MSE* = 415.85, *p* < 0.001, η^2^*_p_* = 0.47. However, exploration time did not differ for participants exploring many long vs. many short chains, (*F* = 0.20 for the between group effect of length-set). No interaction was found (Figure 3). Though participants with a high frequency of long chains had more training material (224 joints vs. 192 joints in total) they were not overall faster. A *t*-test calculated on the average exploration time per joint between the length-set conditions did not reveal any difference, *t*_(17)_ = 0.033, *p* = 0.974.

### Practice function

Quantitative description of practice gains in exploration performance can be captured by fitting a learning curve. The exact shape of the learning curve is relevant, because it is linked to assumptions in theories of skill acquisition (see below and discussion). One distinction linked to qualitative differences in skill acquisition is whether practice related performance gains confirm to the power law or rather to the negative exponential (Heathcote et al., 2000). The most significant difference between the exponential and the power law is the relative learning rate (RLR). While the power function assumes a decreasing RLR, it is constant in the exponential function. A constant RLR means that from trial to trial participants improve performance by the same proportion—relative to the performance gains yet to be reached till the asymptote (e.g., on each trial learn 20% of what remains to be learned). However, many theories of skill acquisition postulated a power law of practice implying a decreasing rather than a constant RLR (Newell and Rosenbloom, 1981; Anderson, 1982; Logan, 1988).

A power law learning curve is modeled by t = A + B ^*^ N^−C^, where *t* is the dependent variable for the time to perform the task, *A* the asymptote, B a constant and *N* the number of practice trials. *C* defines the rate of acceleration with practice. Acceleration is generally negative, i.e., the more trials pass by, the smaller becomes the increase in speed from trial to trial.

**Figure 4:**
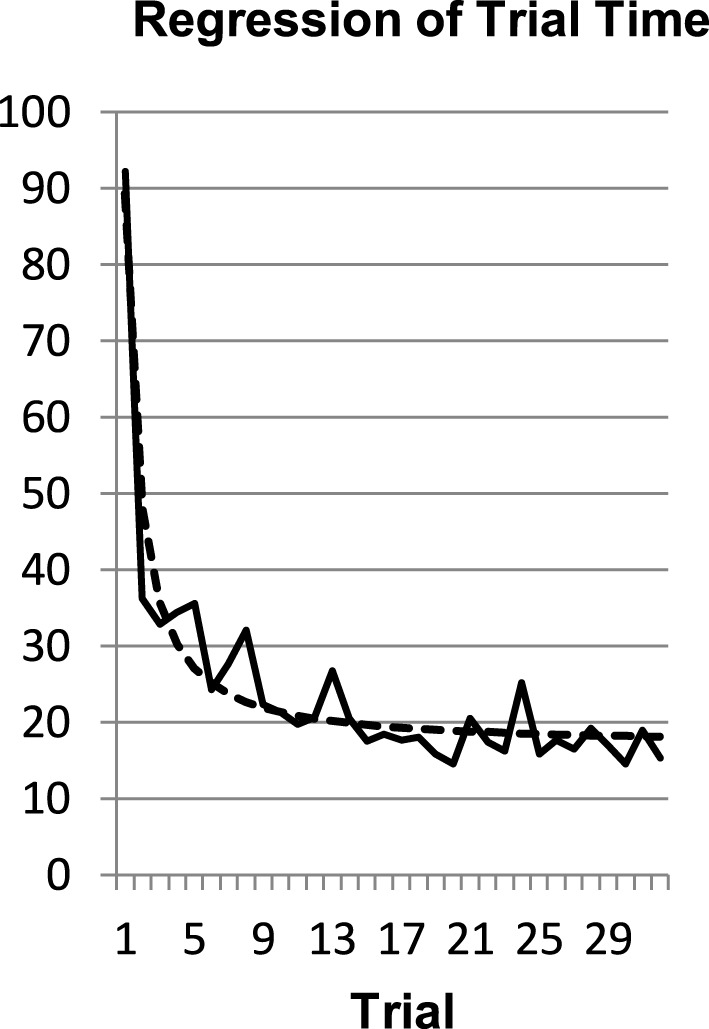
**Averaged data and the power law regression for the exploration time (*R*^2^ = 0.914)**.

To compute the exponential and the power functions we seized the trial-number as the index of practice (*N*) and trial-time as the dependent variables. We used the Levenberg-Marquart estimation in PASW 19. In a first step we checked whether the power function would be an adequate description of the data averaged over participants. The regression for exploration time, *t* = 17.04 + 72.21 ^*^ N^−1.23^, *R*^2^ = 0.914, was described well by a power function regression (Figure 4).

The fit of the power law regression to the averaged exploration time data is very good and better than the exponential fit, *t* = 20.38 + 183.8 ^*^ e^−0.975^*^N^, *R*^2^ = 0.851. However, Heathcote et al. (2000) argued that the regression of averaged data favors the power over the exponential function as a statistical artifact and suggest to compute power and exponential regressions for each participant separately. Indeed, when fitting the data of the participants individually, the average *R*^2^ of the exponential function (*M* = 0.58) was larger than the one of the power function [*M* = 0.46; *t*_(18)_ = 3.286, *p* < 0.01, *d* = 0.73]. This confirms the analysis of Heathcote et al. (2000), who found an advantage of the exponential function over the power function in 33 of 40 different data sets with an average improvement in fit of 17%. Our results and Heathcote et al.'s analysis speak for a constant RLR.

### Components of exploration strategies

After considering the practice function, we wanted to describe the exploration behavior on a more specific level. In a next step we analyzed various specific characteristics of the interactions with the chains in isolation (i.e., the strategy components) in order to lay the ground for combined analyses (i.e., the strategies).

#### Hand vs. hands

The average percentage of right-handed interactions over all participants was 50%, while on average 38% of the interactions were executed with the left hand only and 12% were carried out with both hands simultaneously. A *t*-test confirmed the dominance of the right hand (all participants were right-handed) over the left hand, *t*_(18)_ = 2.98, *p* < 0.01, *d* = 0.66.

#### Use of force

**Figure 5:**
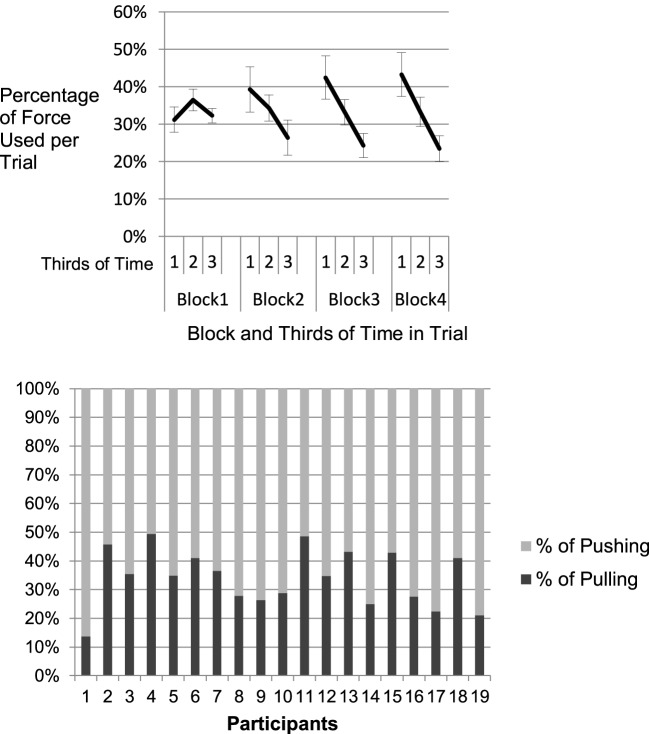
**Use of force within trials and across blocks of practice (upper panel): The x-axis displays thirds of time per trial on the four blocks**. The y-axis shows the percentage of force used on the thirds averaged over all trials of the block, as each block accounts for 100%. The decline of force with proceeding trial-time illustrates the strategy change from constant pushing and pulling in the first block to a more initial use of force at the beginning of the trial. **Lower panel:** All participants show a strong use of pushing, no participant uses pulling as the dominant (>50%) strategy.

Pushing and pulling of the chain required both force and coordinated movement. Typically, in the first trials, participants tended to explore the chains very carefully. Each single joint was explored by an interaction of the adjacent links. Participants switched back and forth between applying force to the chain and marking a joint type. With further experience, in many cases, pushing and pulling was massed at the beginning of each trial. Some participants seemed to aim at bringing the chain into a shape where the type of most of the joints could be identified without interacting with it again during the exploration trial. Joints were then labeled without strong usage of pushing and pulling, just by the visual inspection of the idle structure. Thus, high force in the beginning of a trial should be related to large movements across the chain which could classify the structure of the chain with respect to many joints. Later in the trial, small forces were necessary to explore the remaining joints. For analysis, each trial was separated into three equal time intervals. Force, as the dependent variable was computed as average percentage of force used per third in each block, so that it added up to 100% for each block. Figure 5 (upper panel) suggests that from Block 2 onwards participants allocated the largest percentage of the overall force they applied within a trial to the first third of the trial. This was confirmed by a two factorial repeated measures ANOVA. A significant main effect of time-interval, *F*_(1.28, 23.00)_ = 8.80, *MSE* = 0.053, *p* = 0.004, η^2^_*p*_ = 0.329, and a significant time-interval by block interaction, *F*_(2.99, 53.90)_ = 6.56, *MSE* = 0.014, *p* = 0.001, η^2^_*p*_ = 0.267. An independent effect of block could not be computed since each block added up to 100% of force. The results suggest that at first participants used force at a constant level throughout the trials and then learned to apply force in the beginning of an exploration trial to bring the chain into a shape where the joints could be identified.

#### Sequential interaction with joints

**Figure 6:**
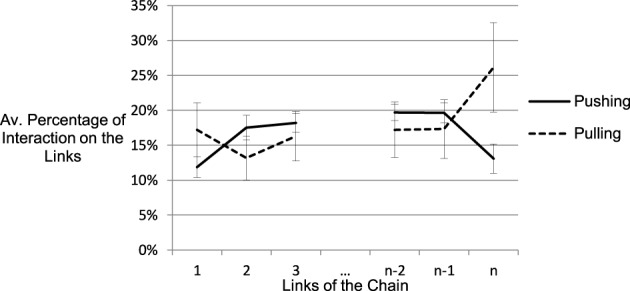
**Percentage of pulling and pushing on the first three and last three links**. Each interaction type over all of the six shown links accounts for 100%. The crossing of the graph illustrates that pulling is used more often at the poles than in the center of the chain, while pushing is mostly used in the center.

To allow for comparisons between the chains of different length, we indexed the position of a link relative to the ends of the chain. Figure 6 shows the relative frequencies for pushing and pulling on the first three and last three links of the chains. The crossing of the two lines illustrates that pushing dominated in the middle part of the chains, while pulling was more prevalent at the outer parts. This behavior was adaptive: Pulling at the ends allowed to stretch the whole chain to reveal the type of many joints while pulling in the middle of the chain would only affect one side of the chain. Conversely, not all links were affected, if the chain was pushed only at one end. Pushing the middle of the chain, however, lead to deformation of the whole structure.

The differences in frequencies are visible in the interaction effect of link position and interaction type, *F*_(3.66, 65.89)_ = 16.48, *MSE* = 65.89, *p* < 0.001, η^2^_*p*_ = 0.48. There were no main effects of link position (*F* = 1.27) and interaction type (*F* = 0.357). Both types of interaction were used on every part of the chain. This fact and the large error bars indicate capacious inter-individual differences and show that the different usage of pushing and pulling on the links is a general tendency rather than a dichotomized, stringent strategy.

#### In which order did participants explore the links of the chains?

**Figure 7:**
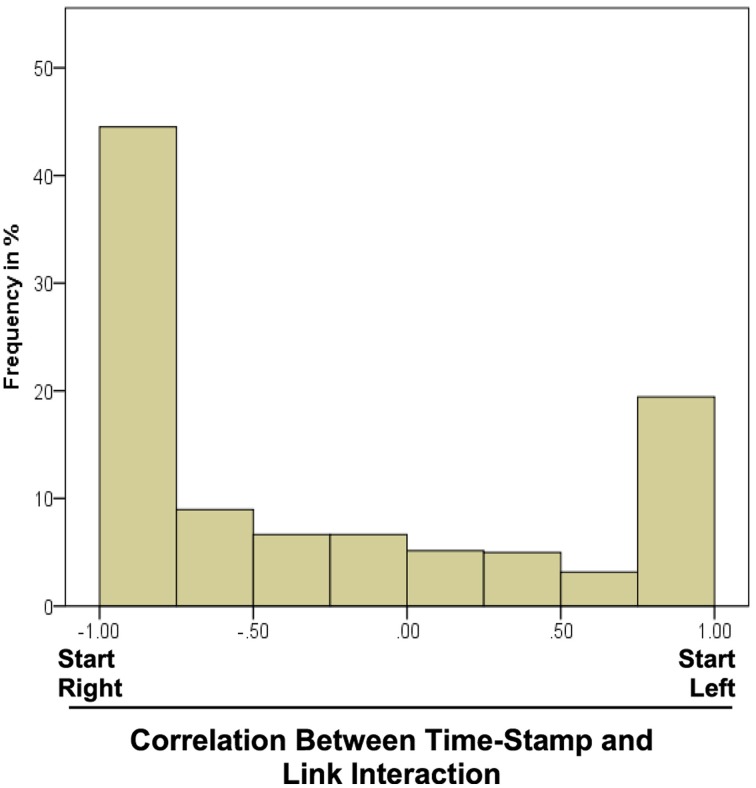
**Histogram of the correlation between time-stamp in the trail and position of the link which was interacted with at that point in time**. Negative values denote a sequential interaction from right to left. Data was averaged over all trials of each participant and then over the participants.

To answers this question, the interaction with the links was analyzed as a function of time. For instance, participants might start pushing or pulling in the middle of the chain and then move outwards. Alternatively, they might explore joint after joint from left to right (or from right to left). In order to quantify the direction and amount of ordering in the sequence, we computed the correlation between (a) the timestamp of the interaction in the trial and (b) the position of the affected joint in the chain individually for each exploration trial in each participant. The positions were numbered from left to right, longer chains ending with higher numbers than shorter chains. In many trials participants either strictly followed the sequential order from right to left or from left to right. The distribution of the correlation over all trials was bimodal (Figure 7). Interaction from left to right in sequence would be reflected in a high positive correlation while negative correlations indicate exploration from right to left. The histogram demonstrates that sequential processing was dominant.

### Evaluating strategy components

The descriptive analysis above helps us to understand how participants explore the kinematic structures and might help to discuss how to improve robotic exploration behavior. However, the diversity of strategic elements will make it difficult to evaluate their advantage unless we relate them to a measure of efficiency of exploration. Sequential processing, the simultaneous use of both hands or the preferences of pushing might differ with respect to how quickly they lead to the discovery of the structure of the chain in a trial. We first report regression analyses linking the different strategy indicators to exploration time per trial. Afterwards we will discuss combinations of strategy indicators that were observed in fast trials. Correlations and regressions were computed over all single trials (i.e., episodes of exploring a single chain) in the data set. As we collected 32 trials from 19 participants, our basis was *N* = 608 trials.

We used a multiple regression with time till completion of exploration in the trial as the criterion variable. If a strategy indicator shows a negative correlation, this indicates that exploration time is the shorter the more evidence for that indicator was obtained in the trial. The multiple regression method allows us to interpret beta weights of the strategy indicators as singular proportions of variance of the criterion. In contrast to a simple correlation analysis the interrelation between the indicators were partialed out in the criterion. To keep track of the gains of the solitary components, a hierarchical setup with four models was applied. To control for the general learning effect, we included the logarithm of the trial-number as the basic predictor. Analogous to the power and exponential function (Figure 4), taking the logarithm leads to a strong effect in the first trials which is reduced with further progress in the experiment. Trial-number's beta therefore should absorb the systematical variance of practice (e.g., getting used to the haptic interface and the task in general). As strategic variables we include (a) the absolute correlation between time-stamp and position of link as an indicator for sequential processing, (b) the average amount of force used on the trial, (c) the percentage of pulling on all interactions (pushing + pulling $\hat{=}$ 100%) and, (d) the percentage of simultaneous use of both hands.

The zero order correlations (Table 1) of all predictors show the isolated role of the trial-number. Log trial-number correlated only weakly with the use of force, *r*_(608)_ = −0.1, *p* = 0.02. This correlation implies the more efficient and therefore reduced use of force during later trials. The high correlation between the simultaneous use of both hands and use of pulling, *r*_(608)_ = 0.40, *p* < 0.001, was also to be expected, because the simultaneous hand use is only an efficient strategy if the chain is stretched through pulling. The additional force which was needed to pull on both sides of the chain was the result of the resistance of the other hand when pulling, and was reflected in the high correlation with the force variable, *r*_(608)_ = 0.63, *p* < 0.001.

**Table 1:** **Zero-order correlations of the five predictors**.

		**1**	**2**	**3**	**4**
1	Trial-number (log)				
2	Sequential Processing	−0.03			
3	% of Pulling	0.05	−0.17^**^		
4	Force	−0.1^*^	−0.11^**^	0.42^**^	
5	Simultaneous Hand-Use	0.07	−0.30^**^	0.40^**^	0.63^**^

* *p < 0.05*,

** *p < 0.01*.

The hierarchical regression (Tables 2, 3) revealed that each predictor is legitimated by a significant increase in R^2^ from model to model, so we will use the most comprehensive Model including all predictors to describe the results of the regression analysis.

**Table 2:** **Coefficents of the hierarchical regression**.

**Model**		**beta**	***t***	**Sig**.	**Collinearity statistics**	
					Tolerance	VIF
1						
	Trial-Number (log)	−0.486	−13.768	*p* <.001	0.999	1.001
	Sequential Processing	−0.145	−4.116	*p* < 0.001	0.999	1.001
2						
	Trial-Number (log)	−0.462	−13.444	*p* < 0.001	0.985	1.015
	Sequential Processing	−0.119	−3.460	*p* < 0.001	0.975	1.025
	Forces	0.224	6.470	*p* < 0.001	0.980	1.021
3						
	Trial-Number (log)	−0.470	−13.701	*p* < 0.001	0.792	1.263
	Sequential Processing	−0.107	−3.084	*p* < 0.007	0.787	1.271
	Forces	0.179	4.692	*p* < 0.001	0.955	1.047
	% of Pulling	0.103	2.699	*p* < 0.001	0.890	1.124
4						
	Trial-Number (log)	−0.455	−13.159	*p* < 0.003	0.778	1.286
	Sequential Processing	−0.135	−3.762	*p* < 0.006	0.493	2.027
	Forces	0.261	5.431	*p* < 0.001	0.999	1.001
	% of Pulling	0.115	2.997	*p* < 0.001	.999	1.001
	Simultaneous Hand-Use	−0.134	−2.778	*p* < 0.001	0.988	1.013

**Table 3:** **Summary for all regression models**.

**Model**	***R^2^***	***R^2^* Change**	**Significance of change**
1	0.254	0.254	*p* < 0.001
2	0.303	0.049	*p* < 0.001
3	0.311	0.008	*p* = 0.007
4	0.32	0.009	*p* = 0.006

The log trial-number (*beta* = −0.455) had the largest beta weight, since it binds the general learning effect. Force has the second strongest weight (*beta* = 0.261). The positive value indicates that a stronger usage of force slows down the exploration process. The converse argument is that a sparse but concentrated use of force, which reveals information about more than one joint, makes faster exploration possible. Compatible with this finding, the simultaneous use of both hands was associated with a faster exploration (*beta* = −0.134). As discussed earlier, this can be explained by the fact that stretching the chain with both virtual hands on the ends, allows to gather information about many joints at the same time. Also, using both hands to push the chain into a shape, where most of the joints could be identified, is a similarly adaptive approach. Yet the general use of pulling was associated positively with the criterion. Since pulling requires more fine-tuned movements, it needs more time (*beta* = 0.115). Sequential processing predicted faster trials (*beta* = −0.135). Likely, we have to take into account that participants were not perfectly adjusted to the handling of the haptic interface after our warm-up phase. The behavior described above might have served to minimize the need for large movements to distant parts of the chain.

While the results have a high face validity and match the informal observations made during the experiment, this regression analysis has several limits. Trials were included in the model without accounting for the variability across participants. However, lending credibility to the current approach, participants did not show highly idiosyncratic strategies. This can be seen in the strong asymmetry of the predictor variables. For instance, the use of both hands was very rare (*Median* = 0.06% of all interactions over all trials) and sequential processing was very common, since the median of absolute correlation values linking the joint index to the exploration order was *r* = 0.91. This constraint does not allow a precise evaluation of the strategy components within participants, because not all possible exploration strategies were applied by each of them. Also it is debatable if speed is the exclusive relevant operationalization for efficiency. The overall force used per trial would also be a reasonable operationalization in some environments. For instance, robots in remote areas (e.g., space robots) would not need to operate fast but power-saving.

Reliability and accordingly error-proneness can also be beheld as measures of the quality of strategies. The more experience the participants gained, the less deformation of the chain was used to mark a joint. A very slight bend was often enough to recognize a joint as revolute. For instance, we observed that participants sometimes tended to mark a joint incorrectly instead of examining it by pushing and pulling. This happened most frequently with revolute joints, falsely marked as rigid because no bend was visible in the shape of the chain. Certainly this error-proneness is associated with strategy components, for instance the intense use of force on every joint expulses misinterpretation on little information about joints.

### Identifying strategies based on components

The multiple regression highlighted the importance of the four different strategy components. Sparse use of force, sequential processing, less pulling behavior and simultaneous hand-use all seem to be adaptive strategy components accelerating exploration behavior. However, only through the combination of the predictors, the definition of strategies becomes possible. For instance, high percentage of pulling is not a strategy for itself, but only a component, which can be included in several strategies. To unfold all strategies, we dichotomized the four predictors by median split and created a table with every possible combination (Table 4, 0 accounting for below median, 1 for above median). The table is ordered by exploration time, so that the most successful strategies are listed first with low strategy numbers. The number of trials which reckon among the pattern of the predictors are displayed, as well as the average trial-number, accounting for the stability and difficulty of the strategy. The average trial-number is computed by averaging the trial-number of all trials assigned to the respective strategies. Therefore, a high number denotes a difficult strategy that was only used in the later trials, while a low number implies simple or ineffective behavior, which was abandoned with further experience. We excluded combinations of predictors, if the average trial-time was not interpretable because of a too small subset (Table 4, crossed cells).

**Table 4:** **Exploration strategies**.

**Strategy-number**	**Sequential processsing**	**Force**	**% of Pulling**	**Simultaneous hand-use**	**Average trial-time in sec**	**CI**	**Average trial-number**	**Number of trials**
1	1	0	0	1	11.94	±1.92	17.33	3
2	0	0	0	1	13.58	±2.19	20.00	29
3	1	0	1	1	14.44	±2.60	19.00	3
4	0	0	1	1	15.12	±4.43	21.86	14
5	1	0	0	0	16.40	±2.20	16.55	115
6	0	0	0	0	17.44	±3.67	16.90	39
7	1	0	1	0	19.70	±2.98	18.23	53
8	1	1	0	0	19.81	±2.78	13.18	39
9	0	1	0	1	20.10	±3.76	17.29	34
10	0	0	1	0	20.79	±5.38	19.66	44
11	1	1	1	0	25.95	±5.01	17.11	38
12	1	1	0	1	26.43	±14.45	17.33	9
13	1	1	1	1	34.42	±6.44	13.20	41
14	0	1	1	0	35.68	±9.58	14.10	29
15	0	1	0	0	36.48	±14.16	11.90	39
16	0	1	1	1	36.57	±9.10	16.36	73

*All combinations of predictors were defined as independent variables and average trial-time, number of trials and average trial-number as dependent variables. The predictors were dichotomized at their median respectively, 0 accounting for below median, 1 for above median*.

The excluded cells (strategies 1 and 3) show that sequential processing does not go along with simultaneous hand use, when sparse force is used. Simultaneous hand use in combination with sparse force is associated with stretching (strategy 3) or pushing (strategy 1) the whole chain to identify all joints at the same time. This process cannot go along with sequential processing, since the chain has to be pulled on both ends at the same time for stretching the whole structure, or pushed in the middle part to reveal all joints. Moving the whole chain in the beginning of the trial, so that the joint types can be directly derived from the shape, is an adaptive and efficient strategy, as discussed before. Strategies 2, 4, and 6 all account for this initial “shaping” and show fast exploration time: Strategy 2 denotes pushing the chain with both hands, strategy 4 stands for pulling the chain with both hands at the ends of the structure and strategy 6 is described by pushing the chain with one device. The average trial-number is rather high for strategies 2 and 4 (20.00, 21.86), indicating comparatively high difficulty.

Another successful strategy was exploring the chain strictly sequentially by interacting with single joints or at least with several parts of the chain successively in sequential order. Strategy 5 depicts this strategy with a low use of force, Strategy 8 with a high use of force. Force is necessary to reveal the properties of the joints, nevertheless a reduced usage of force on sequential processing of the chain is associated with fast exploration (16.40 vs. 19.69 s). Sequential processing can also go along with pulling, though it affords more time to uncover the structure by pulling several joints (Strategy 7) than by pushing them. Strategies 5 and 7 together are used in 168 trials (~28% of all trials), which reflect the strong prevalence of these adaptive sequential strategies.

Slow, non-adaptive strategies are found in the bottom part of the table. The most pronounced gap in trial-time is found between Strategies 12 and 13. While sequential processing with both hands and high percentage of pushing is rather fast (Strategy 12: 26.43 s) the enforced usage of pulling slows down processing by 8 s (Strategy 13: 34.42 s). Strategy 13 can be described as pulling two adjacent links to uncover the interjacent joint. This procedure is repeated on every joint of the chain. Though this strategy helps to avoid any marking errors, it is very slow. The last three strategies are even slower, all applying much force and non-sequential processing. The low average trial-number of Strategies 13–15 (13.2, 14.1, 11.9) indicates that these strategies are not robust and were mainly adopted in the beginning of the experiment, when participants had deficient experience with the simulation environment. The last strategy is similar to number 13, though exploration is not sequential and therefore not as systematic, reflected in even slower exploration time.

Force is not only the strongest predictor in the regression besides the trial-number, but also the only one, which can be evaluated without accounting for interactions. Sparse average use of force generally goes along with faster exploration behavior. However, this average does not reveal how the force is applied best along the trial. Strategies 2, 4, and 6 are associated with “shaping” behavior, a concentrated use of force in the beginning of the exploration. On the other hand Strategies 5, 7, and 8 describe sequential processing with a consistent sparse use of force. These two meta-strategies cannot be combined, since shaping the chain is performed at the ends or in the middle of the chain, but not sequentially along the structure. Notwithstanding both strategic concepts are the most promising ways of exploration. While the shaping strategies seem to be most successful, sequential processing can also be a fast way of exploration, provided that not every joint is explored to carefully with both hands or too much force application (comp. Strategies 12–14). Shaping behavior's effectiveness is fostered by simultaneous hand use (Strategies 2–4) while it is obstructive for sequential processing. Though shaping strategies (2, 4, 6) had a slight advantage over sequential processing in the experiment, they were also more difficult for the participants, as reflected in higher average trial-number. Note that since difficult strategies were applied later in the experiment, they may have in part been executed faster, because participants already had gained more experience with the setup (handling the haptic interface etc.). However, choosing the most efficient strategy is part of the learning progress and accounts for the speeding as well, additional to the adaption to the control of the interfaces.

The descriptive analysis of all strategies (Table 4) revealed the importance of the interaction of the four predictors. We concluded above, that a sparse average use of force is always adaptive, independent of other strategy components. However, the other simple implications of the multiple regression cannot be sustained: The sequential exploration behavior is not *per se* beneficial, because it excludes fast “shaping behavior.” Reduced pulling is also not generally adaptive as the regression data suggested (*beta* = 0.115). Indeed a comparison between strategies 14 and 15 implies an advantage for pulling behavior. The same principle accounts for the last predictor: Data leads to the assumption that simultaneous hand use is impedimental for sequential strategies, while it fosters the effectiveness of “shaping” behavior (compare strategies 2 and 6).

**Figure 8:**
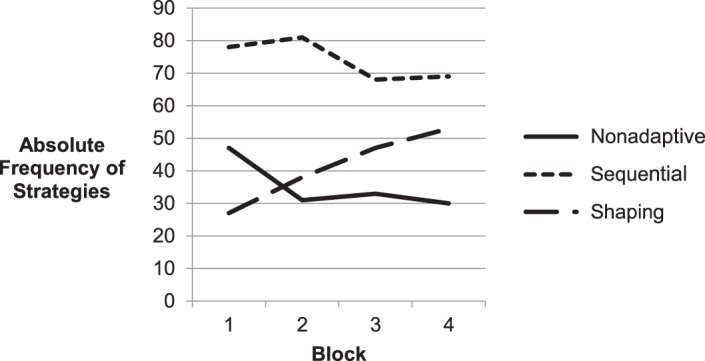
**Absolute frequency of strategies applied on the four training blocks**. While shaping behavior increased constantly over all blocks, the use of non-adaptive strategies decreased after the first block.

The negative Spearman rank correlation between the average trial-number and the strategy-number of all 16 strategies, *r* = −0.63, *p* = 0.008 suggests, that participants learned adaptive but more complex strategies (low strategy-number) with further experience on the experiment (high average trial-number). Non-adaptive strategies, which are characterized by slow and unsystematic exploration behavior would be found in the beginning of the experiment and replaced later by shaping or sequential strategies. To test this assumption, we categorized the strategies as non-adaptive (14, 15, 16), shaping (2, 4, 6, 9, 10) and sequential (5, 7, 8, 11, 12, 13) and tested if the distribution of the strategies' absolute frequencies was dependent of the training block. Since not all participants were able to develop adaptive strategies and their learning progress was very heterogeneous, we decided to test the strategy distribution on the whole sample, not accounting for individual strategy use. The application of shaping strategies increased from 14 trials in Block 1–53 trials in Block 4, while the frequency of sequential strategies was reduced in the second half of the experiment (Figure 8). The accordant chi-square test confirmed that the usage of the strategies was dependent on the experience participants already had gained in the experiment, *X*^2^_(6)_ = 16.21, *p* = 0.013. This analysis fits the negative correlation of strategies' average trial-number and strategy-number and approves our expectation that shaping was the most difficult and most successful strategy. It was developed later in the experiment and used with increasing frequency because of its efficiency. The rather constant level of non-adaptive strategies in Blocks 2–4 might confuse, since we expected a decline with further experience. It can be explained by the computation of the singular strategies. The median split did not allow a discrete distinction of the strategy components so that many non-adaptive strategies might also reflect behavior, which is very similar to sequential or shaping behavior, as its values on the strategies components might be very close to the median.

Again we want to stress that the interpretations involving causality have to be considered with caution. It is possible that the allocation of exploration behavior to the strategies is mediated by the learning of the controllers. The experience with the haptic devices might have led to a reduced usage of force, which changed the value on a strategic component from below median to above median or vice versa. Thus, the experience with the controls, might imply a putative change in strategies, though participants did not change their exploration strategies.

## Discussion

In the current work we studied how humans explore the kinematic structure of chains in a virtual environment by pushing and pulling them bimanually via a haptic interface. In a similar setup Katz et al. (2008) showed that virtual agents are able to gather manipulation knowledge and increase speed in the exploration of chain structures. As a first step toward our goal to combine analytic (humans) and synthetic (robots) study of intelligent exploration, we developed means to quantitatively describe human object exploration. For the first time we could identify and integrate qualitative and quantitative gains in exploration practice in a task tapping cognitive- as well as psycho-motor abilities. We observed large practice-related gains in efficiency in terms of time and force used to classify the kinematic structure of the chains. These learning gains generalized across chains differing in length and composition. Improvements followed the practice function suggested by current work on human skill acquisition. Apart from documenting how much was learned (i.e., the size of the exploration learning effect), we also detailed *what* was learned. On the one hand, we identified strategy components that were associated with efficient (fast) exploration performance: sequential processing, simultaneous use of both hands, low use of pulling rather than pushing, and low use of force. However, only the latter was beneficial irrespective of the characteristics of the other strategy components. Thus, efficient exploration behavior has to be characterized by strategies that simultaneously take into account the abovementioned strategy components.

With practice participants adopted a portfolio of different exploration strategies, allowing for large information gain at the cost of high psycho-motor demands as well as simple-to-execute checking routines. While strategies that were efficient in terms of time and force exerted were used more often with practice, participants did not converge to stereotypical exploration behavior with practice. Neither on the group level, nor on the level of individual participants, the portfolio of strategies was substantially reduced with practice. Rather, participants remained flexible and used multiple exploration ways. In fields as diverse as mental arithmetic in primary school (e.g., Chen and Siegler, 2000), heuristics in multi-reason decision making (e.g., Rieskamp and Otto, 2006), or motor control (e.g., Wolpert and Ghahramani, 2000; Imamizu et al., 2004) probabilistic models of strategy selection have been proposed that lead to practice-related shifts of strategy mixtures rather than to a reduction in strategy variability. For instance, the overlapping waves theory by Siegler and colleagues suggests that old and newly acquired strategies are both used as learning proceeds, so that people can easily fall back on robust and simple calculation strategies in case that newly acquired shortcuts no longer apply. Early on, detailed observations by Muenzinger (1928) have shown that even simple lever presses do not become stereotyped with repeated reinforcement. Applying reinforcement principles on the level of motor routines (e.g., Thorndike, 1898/1911) one could have expected that a guinea pig that (by chance) presses a lever with the right food for the first time will use this food more often, if this motor pattern leads to the delivery of a food pellet. While, according to Muenzinger (1928), the animals did increase their lever press activity, they did not reduce the portfolio on how to press the lever with practice (e.g., continuing to vary between all feet and the noose to press the lever).

We suggest that it is adaptive to keep a large portfolio of strategies and motor patterns to reach a desired end state (i.e., lever quickly pressed for food delivery or structure of chain object quickly classified). Apart from avoiding local minima in the quality strategies used, variability in means might help to reduce the chance that arbitrary aspects of the situation are bound into the learning episode (Ashby et al., 2003; Colzato et al., 2006). Also it might secure a buffer of fallback options to work on a task even when environmental conditions become less stable (poor light, wind, unstable surface) or cognitive and motor resources are reduced by age-related decline or secondary task load (e.g., Verrel et al., 2009). One can speculated that exploration tasks that include psychomotor interactions involving large degrees of freedom in how to reach an exploration goal might help to avoid mechanization in problem solving (Luchins, 1942). While participants working on symbolic paper-and-pencil exploration task tend to stick with the first-best solution that *seems* applicable throughout the set of problems (e.g., Wason, 1960), exploration tasks stressing physical interaction with the object might secure that alternative ways of solving the task are maintained. However, flexibility might come at the cost that redundancies in the composition of the task material are less readily acquired and exploited as compared to setups that place minimal demands on psycho-motor control and offer little potential for variability in performing the task (e.g., Gaschler et al., 2012).

Our results suggest that at the same time participants learned (a) how to operate the haptic input device in the virtual environment and (b) where to affect the chains structures how for revealing the properties of the joints. This might explain the very high gains in performance across the first few trials of practice. Perfect separation between these two aspects of acquisition of exploration skills does not seem reasonable, since some strategies (i.e., bimanual pulling) require highly skilled handling of the interface. We presume that learning of controls is most prominent in the beginning of the experiment, while learning of strategies accrues after some experience with the controls. Compared to many other skill acquisition tasks, the current task was rather complex (cf. Heathcote et al., 2000). The control of the haptic devices was new to the participants and included motor-, sensory,- and cognitive affordances. However, despite task complexity, fitting of practice functions indicated a smooth negative exponential learning process. In line with the decomposition thesis (Lee and Anderson, 2001; Anderson, 2002) this suggests that the practice function summarizing the practice-related performance gains of a complex task does not have to be complex itself. Rather, it can be attributed to the improvement of individual components. One qualitative aspect of learning curves is that they represent the diminishing absolute payoff of practice-investment. From the total performance gain that can be achieved through practice, a large part is yielded by the first few practice units. Later units bring about comparatively modest gains. Exponential and power function have the advantage in common that they offer a quantitative description of practice data in line with this reasoning. Exponential practice functions can be derived from a narrow set of assumptions. As Heathcote et al. (2000) explained, one need only assume that learning is proportional to the time taken to execute the component in case of a continuous mechanism. First, a component that takes longer to execute presents more opportunity for learning. For instance, a slow motor program in service of exploration offers much opportunity for speedup. Second, as learning proceeds, the time to execute the component decreases. Therefore, the absolute learning rate decreases, resulting in exponential learning. Similarly, for discrete mechanisms, such as chunking, exponential learning can be explained by a reduction in learning opportunity. As responses are produced by larger and larger chunks, fewer opportunities for further composition are available. For instance, small motor patterns in service of exploration that are at first executed serially based on online control, become integrated into higher order patterns. Time-demanding control is no longer necessary for small steps but only for scheduling sets consisting of fixed series of small patterns. Naturally, the opportunities for compilation of small single motor programs into larger ones reduce, as more and more patterns are already chunked.

More assumptions are needed in order to theoretically accommodate a decreasing RLR. For instance, Newell and Rosenbloom (1981; see also Anderson, 2002) assumed that chunks are acquired hierarchically and that every time a larger chunk is practiced, this entails practice of its smaller components. Thus, by practicing an exploration motor pattern consisting of single steps, the single steps *and* the overall pattern are fine-tuned. Furthermore, at least in combinatorial environments, acquisition proceeds ordered by chunk span. No larger span chunk is acquired until all chunks of smaller span have been acquired. An exponential learning curve (constant RLR) instead of a power curve (decreasing RLR) would be readily predicted by Newell and Rosenbloom's view on skill acquisition if the second assumption was altered to the stance that chunks are executed as a single unit and therefore practice only themselves, not their constituents. This modification would fit nicely with Newell and Rosenbloom's claim that the execution time for a chunk is independent of its size. Furthermore, Newell and Rosenbloom's theory (even without the latter modification) only predicts a decreasing RLR in case of a combinatorial environment, while otherwise a constant RLR would be predicted (Neves and Anderson, 1981) for the chunking mechanism “composition” producing an exponential practice curve. In a combinatorial learning environment larger chunks are encountered less often than smaller chunks.

In conclusion, the finding that individual participant data are fitted best by the exponential function (while aggregate data are fitted best by the power function) are well in line with work questioning the power law of practice. In each trial participants improved by the same proportion relative to the improvements still possible till the asymptote. Likely, improvements resulted from the fact that individual motor patterns in service of exploration became compiled into larger chunks—not that individual motor patterns received considerable amounts of fine-tuning in addition to the compilation. Potentially, practice phases taking several days rather than 45 min, could have helped to identify both kinds of learning processes. Our results are noteworthy, because our task is much more complex as compared to many skill-acquisition tasks for which this phenomenon has been shown before (compare Heathcote et al., 2000). On the psychomotor level, learning was likely producing performance gains based on improved handling of the haptic interface and haptic and visual processing of the virtual environment. On the cognitive level, participants likely developed knowledge about the kinds of possible joints, their likelihood of occurrence, and their marking color.

The current work provides analytical techniques to describe human exploration behavior quantitatively and qualitatively in a virtual environment that can also be operated by simulated robots. In the future this should allow us to study how efficient human behavior can provide a teaching signal for robot exploration learning and vice versa. Linking human and robot object exploration seems promising as learning and control of exploration behavior can take place on different levels and has complementary weaknesses and strengths. In the simulation study of Katz et al. (2008), training on one chain reduced the number of actions to uncover the structure of another chain by 60% compared to an agent without any experience. These results seem rather impressive, though one has to consider that the virtual robots conducted 50 trials on the training chain before exploring the new chain. Also the new chain, to which the manipulation knowledge was transferred to, was very similar to the initial. When the new chain was not only differing from the initial chain in length but in composition of the joints, the transfer effect was very small. Therefore, the manipulation knowledge transfer seems to be rather specific to the chain material. In contrast, the antecedent analysis of human exploration reveals a far more general effect of training which is even more significant. To compute it, the average exploration time for the 1st trial (no experience) was compared with the average exploration time of the last trial (experience on 31 different chains). The mean reduction in exploration time was 83.5% (CI = 72–90%) in our sample. Participants who worked on many long chains, were not faster on these compared to participants exploring many short and few long chains, so that learning was not specific to the length of the chain, but based on the number of trials already completed. This signifies that human learning processes appear to be more effective and more general than learning in a virtual agent since speeding up was not dependent on the specific attributes of the chains.

Humans followed two different main strategies: (1) Sequential exploration from one end to the other, with several interactions along the chain and (2) Shaping behavior, a massed application of force to the chain either pushing it or stretching it, making nearly all joints definable. After this initial interaction, the joints were marked sequentially. Both of the strategies need only sparse memory capacity and a small focus of attention, as only the movement or the position of the joints just interacted with, are regarded in sequential exploration. When shaping behavior is applied to the chain, the joint type is directly derivable from the rearranged state of the whole structure. For instance, bends in the chain indicate revolute joints, while stretched joints can directly be defined as prismatic.

In contrast, the virtual agent does not analyze the status of the joints in their idle positions, but charts the movement of the chain resulting from the interaction and uses this information to determine the kinematic properties. Therefore, the virtual agent can only gather information while the chain is in motion. The simulated robot analyses all joints simultaneously and has no restriction of focus or memory capacity, so a movement which affects as many joints as possible at one time is adaptive for the robot. It is therefore not surprising that the machine learning algorithm learned to push the chain in the middle, because this reveals information about most of the joints. This robot strategy is comparable to human shaping behavior, as it also should affect the whole chain. However humans gather different information, because they focus on the state of the chain and cannot analyze the movement of all joints simultaneously due to attentional constraints. Notwithstanding, shaping strategies offer robots useful implications for interaction. Strategies 2 and 4 (Table 4) were the most efficient ones in our dataset. Both are shaping strategies comprising simultaneous hand-use. Pulling the chain at both ends or pushing it simultaneously at two contact points should allow the virtual agent to gather a lot of information because this should lead to an expansive movement of the chain as a response to interaction. Besides shaping behavior, sequential exploration is considered as an efficient human strategy. However, it might not be a successful adaption for robotic exploration behavior, since sequential interactions often reveal redundant information about a reduced area of the chain not involving movements of all joints.

With the current work we established the basis for studying human and robot object exploration in a common test bed. We suggest that a perspective joining an analytical (experiments in humans) and a synthetic (designing robot exploration) perspective can advance our understanding of object exploration learning. A joint consideration of psycho-motor as well as cognitive challenges in object exploration seems to be crucial to obtain meaningful results. Furthermore, psycho-motor challenges might be employed to maintain flexibility in object exploration so that agents do not run the risk of binding arbitrary context factors into their learning episodes and are not trapped by local minima in exploration efficiency. The virtual environment allows to flexibly pose such psycho-motor challenges in exploration to humans and to log exploration behavior. On the long run, performance of human and (simulated) robot agents can be compared in the same environment. This offers the potential for human-robot interaction in object exploration as well as for blended learning situations. Robots might be provided with exploration routines as well as heatmaps comprising the relative frequency of human actions in the spatial and temporal space of the exploration environment. Conversely, humans can profit from robot support in dangerous or demanding exploration tasks. In establishing the virtual environment as common test bed for human and robot object exploration, we so far neglected some relevant aspects of virtual environments. The extent to which participants feel presence in the virtual environment has been related to immediate reactions in terms of postural control (e.g., Freeman et al., 2000) and physiological reactions to virtual depth (cf. Insko, 2003). Feeling of presence has been linked to interindividual differences in ignoring vs. using context information to judge orientation (e.g., Hecht and Reiner, 2007). Likely, human performance in object exploration can be boosted by designing the virtual environment such that feeling of presence is high, because participants can rely more on preexisting fast psycho-motor routines in object exploration and less so on effortful online control of actions in the virtual environment. Later research should thus assess and optimize feeling of presence.

### Conflict of interest statement

The authors declare that the research was conducted in the absence of any commercial or financial relationships that could be construed as a potential conflict of interest.
